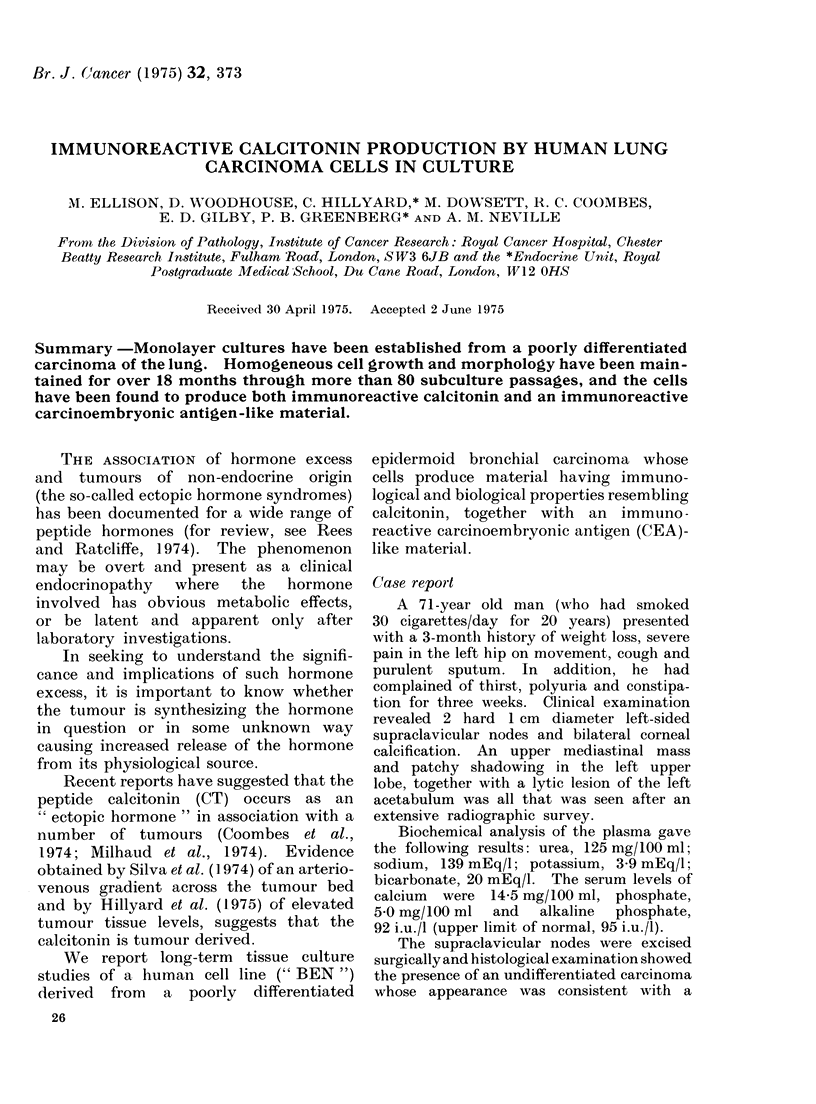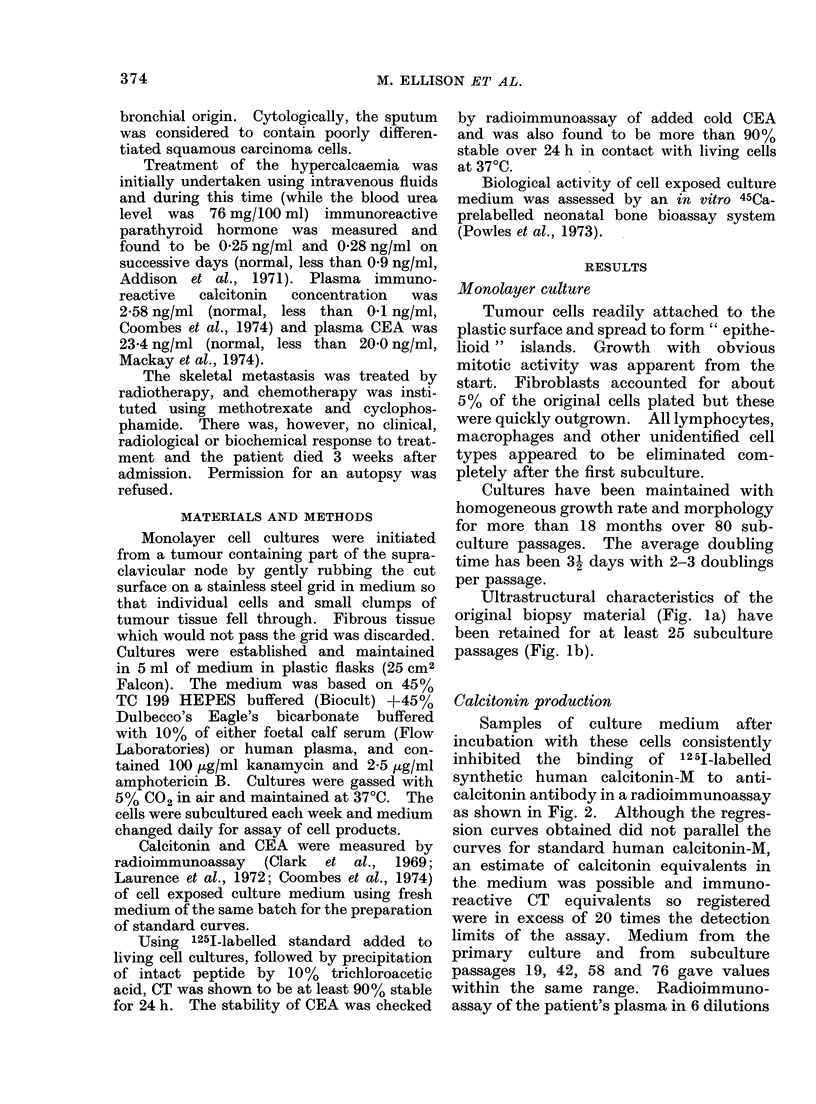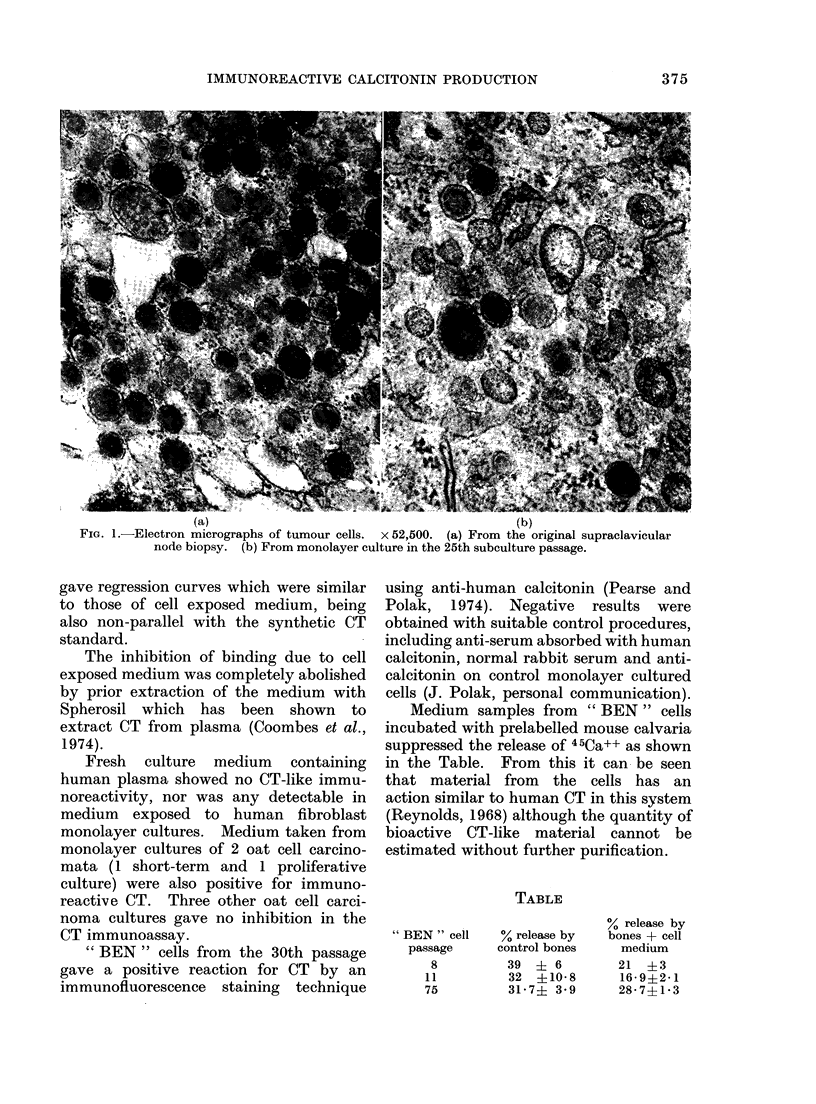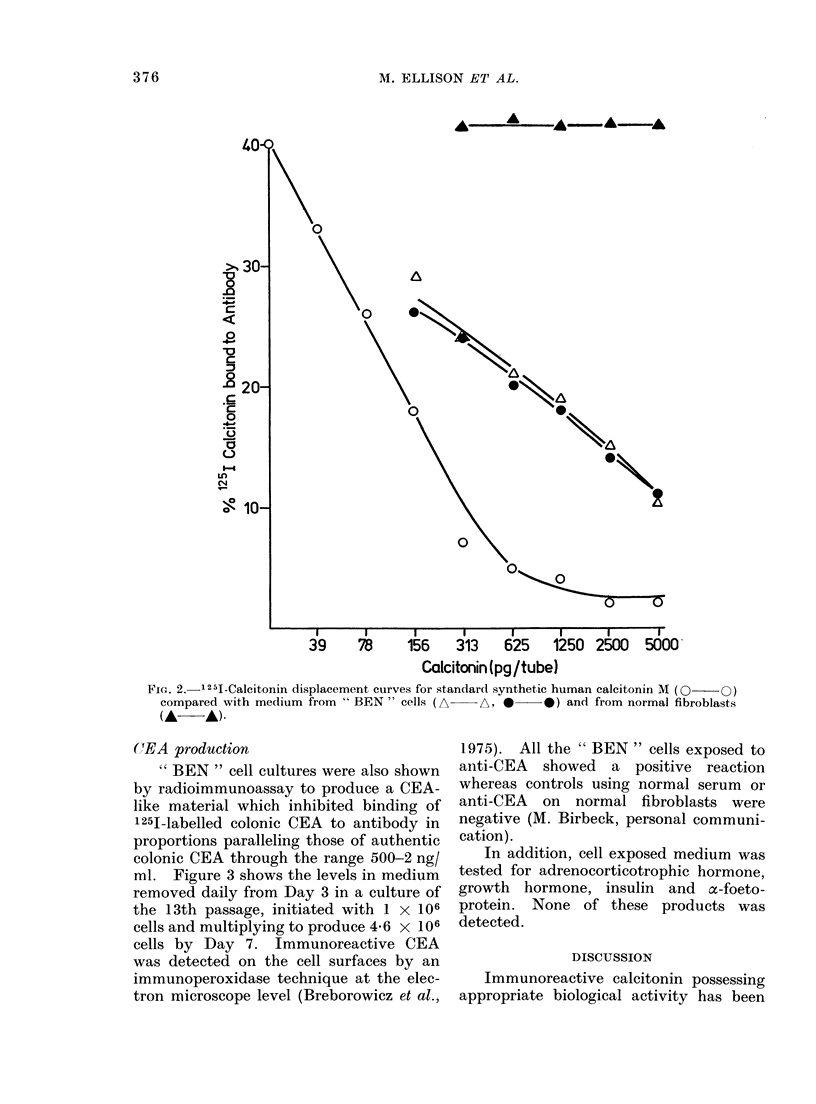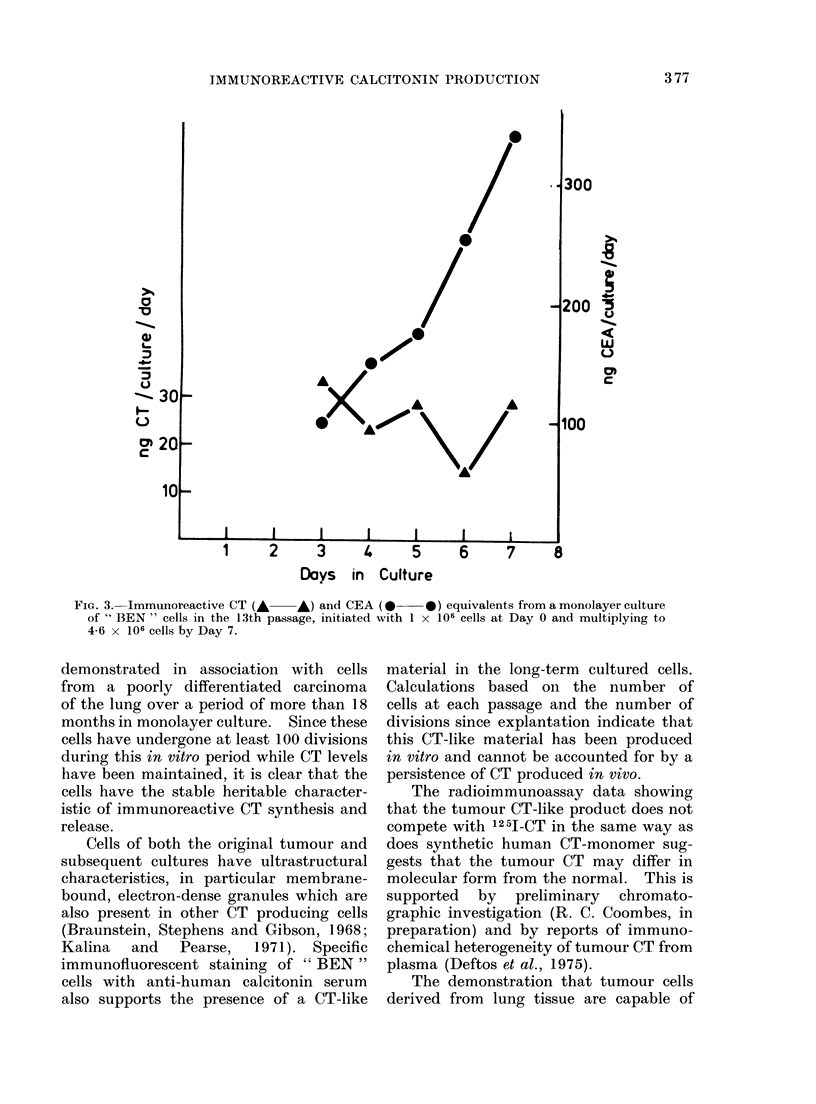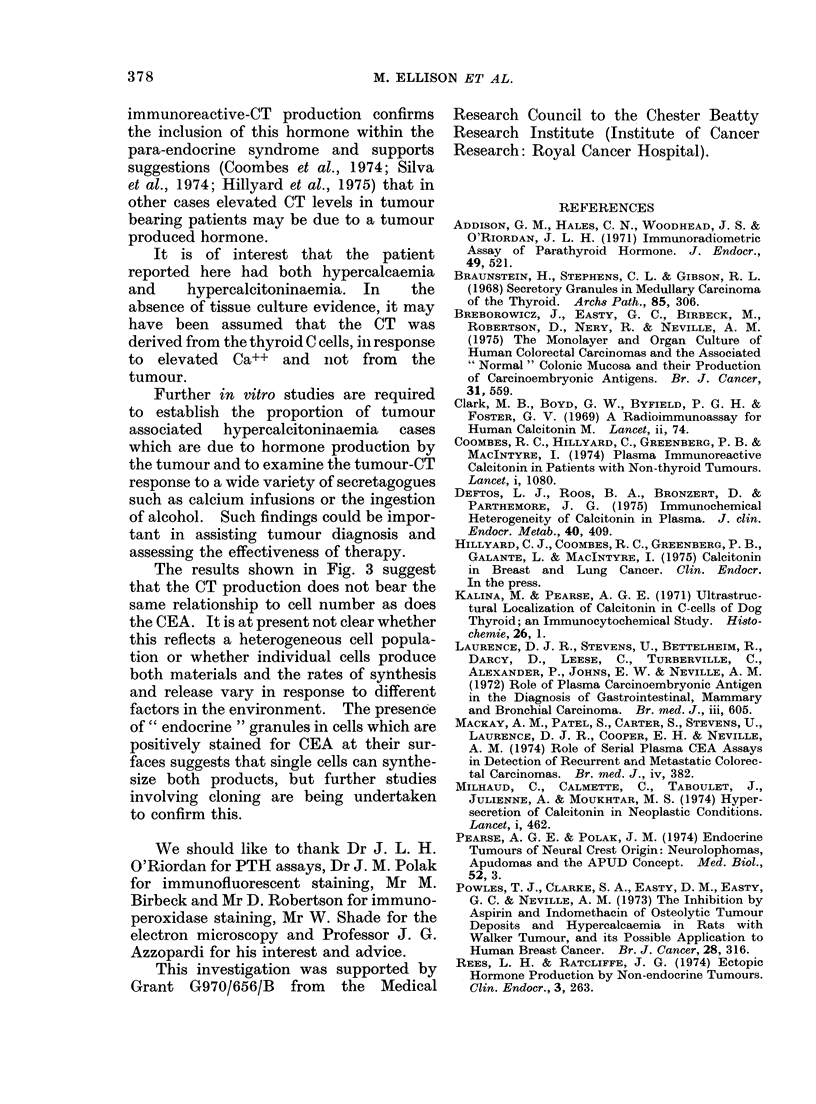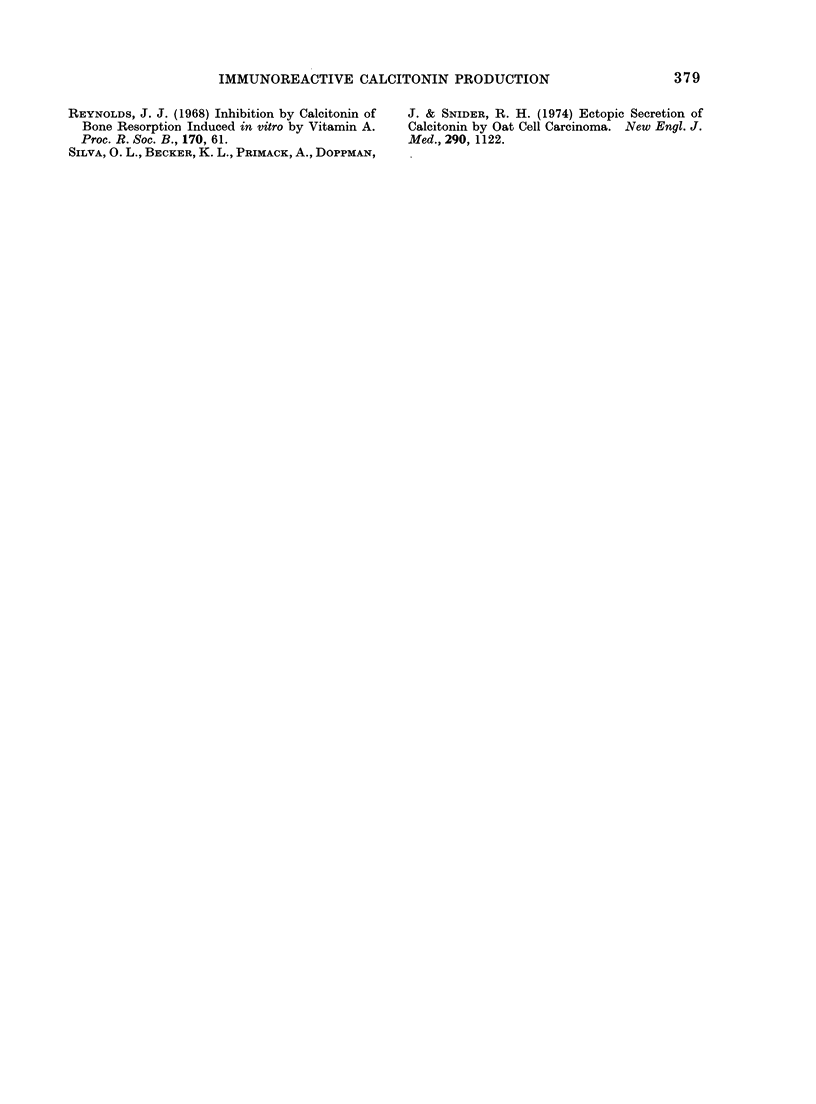# Immunoreactive calcitonin production by human lung carcinoma cells in culture.

**DOI:** 10.1038/bjc.1975.237

**Published:** 1975-09

**Authors:** M. Ellison, D. Woodhouse, C. Hillyard, M. Dowsett, R. C. Coombes, E. D. Gilby, P. B. Greenberg, A. M. Neville

## Abstract

**Images:**


Br. J. Cancer (1975) 32, 373

### IMMUNOREACTIVE CALCITONIN PRODUCTION BY HUMAN LUNG CARCINOMA CELLS IN CULTURE

M. ELLISON, D. WOODHOUSE, C. HILLYARD,* M. DOWSETT, R. C. COOMBES, E. D. GILBY, P. B. GREENBERG* AND A. M. NEVILLE

*From the Division of Pathology, Institute of Cancer Research: Royal Cancer Hospital, Chester Beatty Research Institute, Fulham Road, London, SW3 6JB and the *Endocrine Unit, Royal Postgraduate Medical School, Du Cane Road, London, W12 0HS*

Received 30 April 1975. Accepted 2 June 1975

**Summary —Monolayer cultures have been established from a poorly differentiated carcinoma of the lung. Homogeneous cell growth and morphology have been maintained for over 18 months through more than 80 subculture passages, and the cells have been found to produce both immunoreactive calcitonin and an immunoreactive carcinoembryonic antigen-like material.**

THE ASSOCIATION of hormone excess and tumours of non-endocrine origin (the so-called ectopic hormone syndromes) has been documented for a wide range of peptide hormones (for review, see Rees and Ratcliffe, 1974). The phenomenon may be overt and present as a clinical endocrinopathy where the hormone involved has obvious metabolic effects, or be latent and apparent only after laboratory investigations.

In seeking to understand the significance and implications of such hormone excess, it is important to know whether the tumour is synthesizing the hormone in question or in some unknown way causing increased release of the hormone from its physiological source.

Recent reports have suggested that the peptide calcitonin (CT) occurs as an " ectopic hormone " in association with a number of tumours (Coombes *et al.*, 1974; Milhaud *et al.*, 1974). Evidence obtained by Silva *et al.* (1974) of an arteriovenous gradient across the tumour bed and by Hillyard *et al.* (1975) of elevated tumour tissue levels, suggests that the calcitonin is tumour derived.

We report long-term tissue culture studies of a human cell line (" BEN ") derived from a poorly differentiated epidermoid bronchial carcinoma whose cells produce material having immunological and biological properties resembling calcitonin, together with an immunoreactive carcinoembryonic antigen (CEA)-like material.

### *Case report*

A 71-year old man (who had smoked 30 cigarettes/day for 20 years) presented with a 3-month history of weight loss, severe pain in the left hip on movement, cough and purulent sputum. In addition, he had complained of thirst, polyuria and constipation for three weeks. Clinical examination revealed 2 hard 1 cm diameter left-sided supraclavicular nodes and bilateral corneal calcification. An upper mediastinal mass and patchy shadowing in the left upper lobe, together with a lytic lesion of the left acetabulum was all that was seen after an extensive radiographic survey.

Biochemical analysis of the plasma gave the following results: urea, 125 mg/100 ml; sodium, 139 mEq/l; potassium, 3·9 mEq/l; bicarbonate, 20 mEq/l. The serum levels of calcium were 14·5 mg/100 ml, phosphate, 5·0 mg/100 ml and alkaline phosphate, 92 i.u./l (upper limit of normal, 95 i.u./l).

The supraclavicular nodes were excised surgically and histological examination showed the presence of an undifferentiated carcinoma whose appearance was consistent with a bronchial origin. Cytologically, the sputum was considered to contain poorly differentiated squamous carcinoma cells.

Treatment of the hypercalcaemia was initially undertaken using intravenous fluids and during this time (while the blood urea level was 76 mg/100 ml) immunoreactive parathyroid hormone was measured and found to be 0·25 ng/ml and 0·28 ng/ml on successive days (normal, less than 0·9 ng/ml, Addison *et al.*, 1971). Plasma immunoreactive calcitonin concentration was 2·58 ng/ml (normal, less than 0·1 ng/ml, Coombes *et al.*, 1974) and plasma CEA was 23·4 ng/ml (normal, less than 20·0 ng/ml, Mackay *et al.*, 1974).

The skeletal metastasis was treated by radiotherapy, and chemotherapy was instituted using methotrexate and cyclophosphamide. There was, however, no clinical, radiological or biochemical response to treatment and the patient died 3 weeks after admission. Permission for an autopsy was refused.

### MATERIALS AND METHODS

Monolayer cell cultures were initiated from a tumour containing part of the supraclavicular node by gently rubbing the cut surface on a stainless steel grid in medium so that individual cells and small clumps of tumour tissue fell through. Fibrous tissue which would not pass the grid was discarded. Cultures were established and maintained in 5 ml of medium in plastic flasks (25 $cm^2$ Falcon). The medium was based on 45% TC 199 HEPES buffered (Biocult) +45% Dulbecco's Eagle's bicarbonate buffered with 10% of either foetal calf serum (Flow Laboratories) or human plasma, and contained 100 $\mu$g/ml kanamycin and 2·5 $\mu$g/ml amphotericin B. Cultures were gassed with 5% $CO_2$ in air and maintained at 37°C. The cells were subcultured each week and medium changed daily for assay of cell products.

Calcitonin and CEA were measured by radioimmunoassay (Clark *et al.*, 1969; Laurence *et al.*, 1972; Coombes *et al.*, 1974) of cell exposed culture medium using fresh medium of the same batch for the preparation of standard curves.

Using $^{125}$I-labelled standard added to living cell cultures, followed by precipitation of intact peptide by 10% trichloroacetic acid, CT was shown to be at least 90% stable for 24 h. The stability of CEA was checked by radioimmunoassay of added cold CEA and was also found to be more than 90% stable over 24 h in contact with living cells at 37°C.

Biological activity of cell exposed culture medium was assessed by an *in vitro* $^{45}$Ca-prelabelled neonatal bone bioassay system (Powles *et al.*, 1973).

### RESULTS

### *Monolayer culture*

Tumour cells readily attached to the plastic surface and spread to form "epithelioid" islands. Growth with obvious mitotic activity was apparent from the start. Fibroblasts accounted for about 5% of the original cells plated but these were quickly outgrown. All lymphocytes, macrophages and other unidentified cell types appeared to be eliminated completely after the first subculture.

Cultures have been maintained with homogeneous growth rate and morphology for more than 18 months over 80 subculture passages. The average doubling time has been $3\frac{1}{2}$ days with 2–3 doublings per passage.

Ultrastructural characteristics of the original biopsy material (Fig. 1a) have been retained for at least 25 subculture passages (Fig. 1b).

### *Calcitonin production*

Samples of culture medium after incubation with these cells consistently inhibited the binding of $^{125}$I-labelled synthetic human calcitonin-M to anti-calcitonin antibody in a radioimmunoassay as shown in Fig. 2. Although the regression curves obtained did not parallel the curves for standard human calcitonin-M, an estimate of calcitonin equivalents in the medium was possible and immunoreactive CT equivalents so registered were in excess of 20 times the detection limits of the assay. Medium from the primary culture and from subculture passages 19, 42, 58 and 76 gave values within the same range. Radioimmunoassay of the patient's plasma in 6 dilutions

(a) (b)

FIG. 1.—Electron micrographs of tumour cells. ×52,500. (a) From the original supraclavicular node biopsy. (b) From monolayer culture in the 25th subculture passage.

gave regression curves which were similar to those of cell exposed medium, being also non-parallel with the synthetic CT standard.

The inhibition of binding due to cell exposed medium was completely abolished by prior extraction of the medium with Spherosil which has been shown to extract CT from plasma (Coombes *et al.*, 1974).

Fresh culture medium containing human plasma showed no CT-like immunoreactivity, nor was any detectable in medium exposed to human fibroblast monolayer cultures. Medium taken from monolayer cultures of 2 oat cell carcinomata (1 short-term and 1 proliferative culture) were also positive for immunoreactive CT. Three other oat cell carcinoma cultures gave no inhibition in the CT immunoassay.

" BEN " cells from the 30th passage gave a positive reaction for CT by an immunofluorescence staining technique using anti-human calcitonin (Pearse and Polak, 1974). Negative results were obtained with suitable control procedures, including anti-serum absorbed with human calcitonin, normal rabbit serum and anti-calcitonin on control monolayer cultured cells (J. Polak, personal communication).

Medium samples from " BEN " cells incubated with prelabelled mouse calvaria suppressed the release of $^{45}Ca^{++}$ as shown in the Table. From this it can be seen that material from the cells has an action similar to human CT in this system (Reynolds, 1968) although the quantity of bioactive CT-like material cannot be estimated without further purification.

TABLE

| " BEN " cell passage | % release by control bones | % release by bones + cell medium |
|---|---|---|
| 8 | 39 ± 6 | 21 ±3 |
| 11 | 32 ±10·8 | 16·9±2·1 |
| 75 | 31·7± 3·9 | 28·7±1·3 |

FIG. 2.—$^{125}$I-Calcitonin displacement curves for standard synthetic human calcitonin M (○——○) compared with medium from " BEN " cells (△——△, ●——●) and from normal fibroblasts (▲——▲).

### CEA production

" BEN " cell cultures were also shown by radioimmunoassay to produce a CEA-like material which inhibited binding of $^{125}$I-labelled colonic CEA to antibody in proportions paralleling those of authentic colonic CEA through the range 500–2 ng/ml. Figure 3 shows the levels in medium removed daily from Day 3 in a culture of the 13th passage, initiated with $1 \times 10^6$ cells and multiplying to produce $4 \cdot 6 \times 10^6$ cells by Day 7. Immunoreactive CEA was detected on the cell surfaces by an immunoperoxidase technique at the electron microscope level (Breborowicz *et al.*, 1975). All the " BEN " cells exposed to anti-CEA showed a positive reaction whereas controls using normal serum or anti-CEA on normal fibroblasts were negative (M. Birbeck, personal communication).

In addition, cell exposed medium was tested for adrenocorticotrophic hormone, growth hormone, insulin and α-foetoprotein. None of these products was detected.

### DISCUSSION

Immunoreactive calcitonin possessing appropriate biological activity has been

FIG. 3.—Immunoreactive CT (▲——▲) and CEA (●——●) equivalents from a monolayer culture of " BEN " cells in the 13th passage, initiated with 1 × $10^6$ cells at Day 0 and multiplying to 4·6 × $10^6$ cells by Day 7.

demonstrated in association with cells from a poorly differentiated carcinoma of the lung over a period of more than 18 months in monolayer culture. Since these cells have undergone at least 100 divisions during this *in vitro* period while CT levels have been maintained, it is clear that the cells have the stable heritable characteristic of immunoreactive CT synthesis and release.

Cells of both the original tumour and subsequent cultures have ultrastructural characteristics, in particular membrane-bound, electron-dense granules which are also present in other CT producing cells (Braunstein, Stephens and Gibson, 1968; Kalina and Pearse, 1971). Specific immunofluorescent staining of " BEN " cells with anti-human calcitonin serum also supports the presence of a CT-like material in the long-term cultured cells. Calculations based on the number of cells at each passage and the number of divisions since explantation indicate that this CT-like material has been produced *in vitro* and cannot be accounted for by a persistence of CT produced *in vivo*.

The radioimmunoassay data showing that the tumour CT-like product does not compete with $^{125}$I-CT in the same way as does synthetic human CT-monomer suggests that the tumour CT may differ in molecular form from the normal. This is supported by preliminary chromatographic investigation (R. C. Coombes, in preparation) and by reports of immunochemical heterogeneity of tumour CT from plasma (Deftos *et al.*, 1975).

The demonstration that tumour cells derived from lung tissue are capable of immunoreactive-CT production confirms the inclusion of this hormone within the para-endocrine syndrome and supports suggestions (Coombes *et al.*, 1974; Silva *et al.*, 1974; Hillyard *et al.*, 1975) that in other cases elevated CT levels in tumour bearing patients may be due to a tumour produced hormone.

It is of interest that the patient reported here had both hypercalcaemia and hypercalcitoninaemia. In the absence of tissue culture evidence, it may have been assumed that the CT was derived from the thyroid C cells, in response to elevated $Ca^{++}$ and not from the tumour.

Further *in vitro* studies are required to establish the proportion of tumour associated hypercalcitoninaemia cases which are due to hormone production by the tumour and to examine the tumour-CT response to a wide variety of secretagogues such as calcium infusions or the ingestion of alcohol. Such findings could be important in assisting tumour diagnosis and assessing the effectiveness of therapy.

The results shown in Fig. 3 suggest that the CT production does not bear the same relationship to cell number as does the CEA. It is at present not clear whether this reflects a heterogeneous cell population or whether individual cells produce both materials and the rates of synthesis and release vary in response to different factors in the environment. The presence of " endocrine " granules in cells which are positively stained for CEA at their surfaces suggests that single cells can synthesize both products, but further studies involving cloning are being undertaken to confirm this.

We should like to thank Dr J. L. H. O'Riordan for PTH assays, Dr J. M. Polak for immunofluorescent staining, Mr M. Birbeck and Mr D. Robertson for immunoperoxidase staining, Mr W. Shade for the electron microscopy and Professor J. G. Azzopardi for his interest and advice.

This investigation was supported by Grant G970/656/B from the Medical Research Council to the Chester Beatty Research Institute (Institute of Cancer Research: Royal Cancer Hospital).

### REFERENCES

ADDISON, G. M., HALES, C. N., WOODHEAD, J. S. & O'RIORDAN, J. L. H. (1971) Immunoradiometric Assay of Parathyroid Hormone. *J. Endocr.*, **49**, 521.

BRAUNSTEIN, H., STEPHENS, C. L. & GIBSON, R. L. (1968) Secretory Granules in Medullary Carcinoma of the Thyroid. *Archs Path.*, **85**, 306.

BREBOROWICZ, J., EASTY, G. C., BIRBECK, M., ROBERTSON, D., NERY, R. & NEVILLE, A. M. (1975) The Monolayer and Organ Culture of Human Colorectal Carcinomas and the Associated " Normal " Colonic Mucosa and their Production of Carcinoembryonic Antigens. *Br. J. Cancer*, **31**, 559.

Clark, M. B., BOYD, G. W., BYFIELD, P. G. H. & FOSTER, G. V. (1969) A Radioimmunoassay for Human Calcitonin M. *Lancet*, ii, 74.

COOMBES, R. C., HILLYARD, C., GREENBERG, P. B. & MACINTYRE, I. (1974) Plasma Immunoreactive Calcitonin in Patients with Non-thyroid Tumours. *Lancet*, i, 1080.

DEFTOS, L. J., ROOS, B. A., BRONZERT, D. & PARTHEMORE, J. G. (1975) Immunochemical Heterogeneity of Calcitonin in Plasma. *J. clin. Endocr. Metab.*, **40**, 409.

HILLYARD, C. J., COOMBES, R. C., GREENBERG, P. B., GALANTE, L. & MACINTYRE, I. (1975) Calcitonin in Breast and Lung Cancer. *Clin. Endocr.* In the press.

KALINA, M. & PEARSE, A. G. E. (1971) Ultrastructural Localization of Calcitonin in C-cells of Dog Thyroid; an Immunocytochemical Study. *Histochemie*, **26**, 1.

LAURENCE, D. J. R., STEVENS, U., BETTELHEIM, R., DARCY, D., LEESE, C., TURBERVILLE, C., ALEXANDER, P., JOHNS, E. W. & NEVILLE, A. M. (1972) Role of Plasma Carcinoembryonic Antigen in the Diagnosis of Gastrointestinal, Mammary and Bronchial Carcinoma. *Br. med. J.*, iii, 605.

MACKAY, A. M., PATEL, S., CARTER, S., STEVENS, U., LAURENCE, D. J. R., COOPER, E. H. & NEVILLE, A. M. (1974) Role of Serial Plasma CEA Assays in Detection of Recurrent and Metastatic Colorectal Carcinomas. *Br. med. J.*, iv, 382.

MILHAUD, C., CALMETTE, C., TABOULET, J., JULIENNE, A. & MOUKHTAR, M. S. (1974) Hypersecretion of Calcitonin in Neoplastic Conditions. *Lancet*, i, 462.

PEARSE, A. G. E. & POLAK, J. M. (1974) Endocrine Tumours of Neural Crest Origin: Neurolophomas, Apudomas and the APUD Concept. *Med. Biol.*, **52**, 3.

POWLES, T. J., CLARKE, S. A., EASTY, D. M., EASTY, G. C. & NEVILLE, A. M. (1973) The Inhibition by Aspirin and Indomethacin of Osteolytic Tumour Deposits and Hypercalcaemia in Rats with Walker Tumour, and its Possible Application to Human Breast Cancer. *Br. J. Cancer*, **28**, 316.

REES, L. H. & RATCLIFFE, J. G. (1974) Ectopic Hormone Production by Non-endocrine Tumours. *Clin. Endocr.*, **3**, 263.

REYNOLDS, J. J. (1968) Inhibition by Calcitonin of Bone Resorption Induced *in vitro* by Vitamin A. *Proc. R. Soc. B.*, **170**, 61.

SILVA, O. L., BECKER, K. L., PRIMACK, A., DOPPMAN, J. & SNIDER, R. H. (1974) Ectopic Secretion of Calcitonin by Oat Cell Carcinoma. *New Engl. J. Med.*, **290**, 1122.